# Determinants of handwashing behaviour among primary school teachers in a district of Ghana

**DOI:** 10.1080/21642850.2023.2185620

**Published:** 2023-03-15

**Authors:** Samson Gbolu, Emmanuel Appiah-Brempong, Paul Okyere, Hasehni Vampere, Gloria Obeng Nyarko, Kofi Akohene Mensah

**Affiliations:** a Department of Health Promotion and Disability Studies, School of Public Health, Kwame Nkrumah Uuiversity of Science and Technology (KNUST), Kumasi, Ghana; b Department of Health Policy, Management and Economics, School of Public Health, Kwame Nkrumah Uuiversity of Science and Technology (KNUST), Kumasi, Ghana

**Keywords:** Handwashing determinants, handwashing with soap, teachers’ handwashing behaviour, hygiene, Ghana

## Abstract

**Background:** Hand hygiene practise is an effective school-based measure for infectious disease prevention, especially in developing countries. School children model their behaviour through the observation of significant others, including teachers. However, little is known about the handwashing behaviour and factors influencing the handwashing practises of teachers at the primary school level in Ghana.

**Methods:** A quantitative cross-sectional study was conducted among 214 primary school teachers, recruited by convenience sampling. Data were collected using a structured questionnaire which were designed based on selected constructs in the Theory of Planned Behaviour and the Health Belief Model. Data analyses was done with the aid of STATA software, version 14.0. To identify determinants of hand washing with soap (HWWS) among participants, correlation and multiple linear regression analysis were used.

**Results:** The participants’ mean SD age was 34.7 7.6 years, ranging from 20 to 51 years. The majority were males (87.9%). The majority (84.0% and 86.0%) of the teachers, respectively, reported HWWS practises after using the toilet and before eating with bare hands. A correlation was found between reported HWWS practise and toilet use (*r* = 0.64; *p* = 0.001) and eating with bare hands (*r* = 0.84; *p* = 0.001). A multiple linear regression analysis found that knowledge (*p* = 0.001), attitude toward HWWS (*p* = 0.002), and teachers’ perception of the severity of diarrhoea (*p* = 0.009) were determinants of teachers’ reported HWWS behaviours.

**Conclusion:** Teachers’ perceptions of their susceptibility to and severity of diarrhoea, and their knowledge and attitude towards HWWS at critical times should be focus areas for handwashing programmes to achieve the desired outcomes.

## BACKGROUND

Handwashing with soap (HWWS) under running water is noted to be a simple yet effective health-promoting practise that could reduce or eliminate germs (Mbakaya, 2022)). Access to water, sanitation and hygiene (WASH) is a human right, yet billions are still faced with daily challenges accessing even the most basic of services such as handwashing stations (UNDP, 2018). Management of sanitation and hygiene services remains a global challenge, particularly in the school setting (Chatterley et al., 2014). Poor hygiene and sanitation are major problems in developing countries and remain high-risk behaviours in primary schools (Assefa & Kumie, 2014). This can be managed by providing handwashing facilities or initiatives in primary schools, which have been identified to increase HWWS rates among both teachers and pupils (USAID, 2008; Chittleborough et al., 2013; Sohn et al., 2012).

In Ghana, a significant number of people do not have access to sanitation facilities, and this is not different from the sanitation situation in schools, especially primary schools (Akpakli et al., 2018). According to the Multiple Indicator Cluster Survey (2017/2018), only one out of every five Ghanaian households has improved sanitation facilities such as septic tanks or pit latrines, flush/pour flush pipe sewer system, composting toilets, or ventilated improved pit latrines (Ghana Statistical Service, 2019). Where improved sanitation and handwashing facilities are readily available, visible, and easy to reach, it enhances one’s handwashing behaviour (Suresh & Cahill, 2007). In primary school settings, pupils model their behaviour and attitude after their teachers and are also expected to develop a good reasoning habit for making informed choices (Acar et al., 2018; Frenzel et al., 2018; and Diezmann et al., 2002). The Bandura’s Social Learning Theory has it that human behaviour is learned by observing, imitating, and modelling that of others (Bandura, 2001). This makes the teacher’s hand hygiene behaviour an integral component in promoting positive hand hygiene behaviour among primary school pupils. Snow et al. (2008), cited by Appiah-Brempong et al. (2017), affirm that a teacher’s cue to action in combination with hand hygiene educational intervention is effective in promoting hand hygiene behaviour among school pupils.

Today, in low-socioeconomic countries, no or unsafe handwashing practises are still a major contributing factor to the burden of diseases (Shrestha et al., 2018). Although HWWS is cost-effective and easy to practice, its adherence at critical times remains low among various communities (Saboori et al., 2013). Studies that investigated the determinants of people’s handwashing behaviour reported some level of positive relationship between knowledge or information on hand hygiene and the practise of HWWS (Chittleborough et al., 2013; Global Handwashing Partnership, 2018).

Shmidt et al.'s (2009) cross-sectional study in Kenyan households revealed that HWWS was practised more after feacal contact (32%) than before food handling (15%). This behaviour was statistically associated with the participants’ educational level, access to water, and exposure to hand hygiene information (ibid.). Other predictors of HWWS were identified as the motivation for personal hygiene, a habitual practice, and a lack of concern for the cost of soap (Aunger et al., 2010). A systematic review by White et al. (2020) reported that the commonly mentioned predictors of HWWS were knowledge, perceived risk of infection, educational level, wealth, availability of WASH infrastructure, and gender of the participants.

Ravichandran et al. (2019) study in a medical college in India, and Appiah-Brempong et al. (2020) study among primary school children in Ghana showed that knowledge of hand hygiene may not necessarily translate into handwashing behaviour nor positive intentions or attitudes toward handwashing. Even though the knowledge of hand hygiene is known to influence proper handwashing behaviour, the fear of disease infection (perceived susceptibility) and its severity (perceived severity) largely determines the compliance to hygiene practices, even in the midst of barriers (Gillespie, 2013). Appiah-Brempong et al. (2017) therefore recommended that hand hygiene intervention in schools should target individual variables such as the knowledge and skills of the target group on hand hygiene, their perceived susceptibility to infections and their severity, attitude, and subjective norms (normative beliefs and the motivation to comply with the hand hygiene behaviour). Even though few studies investigated teachers’ handwashing behaviour, their crucial role in influencing the handwashing behaviour of school children has been acknowledged (Setyautami et al., 2012). Hand hygiene interventions in schools must therefore include the teacher as a subjective normative belief predictor of children's handwashing behaviour in the school setting (Appiah-Brempong et al., 2017). It is therefore worth investigating the hand-washing behaviour of primary school teachers so that intervention effects are maximised; especially in rural settings where such studies appear scanty.

This study examined primary school teachers’ knowledge of handwashing, their reported handwashing behaviour and its determinants in the context of selected constructs in the Theory of Planned Behavior (TPB) – attitude and the Health Belief Model (HBM) – perceived susceptibility and perceived severity in the Sene East district of Ghana. These constructs have been found to be critical in predicting health-related behaviours. Teachers’ decision to practice handwashing behaviour is largely dependent upon on their knowledge and attitude towards handwashing and their perceived susceptibility to infections like diarrhoea diseases and its severity.

## Methods

### Study design

The study adopted a descriptive cross-sectional design and a quantitative approach. This approach and design allow for an objective investigation into teachers’ handwashing behaviour, resulting in results that are generalisable, especially in settings with similar characteristics.

### Study setting

The study was conducted in the Sene East District in the Bono East Region of Ghana. The district has 36 kindergarten schools, 49 primary schools, 17 junior high schools, and one senior high school. The district has five administrative circuits based on the Ghana Education Service demarcations. The primary school level has a total of 217 teachers. The 2020 District League Table (DLT), which assessed and ranked districts based on disparities in resource allocation and development, ranked the Sene East district among the ten (10) lowest scoring districts in water and sanitation out of the 260 districts in Ghana (National Development Planning Commission (NDPC) and UNICEF Ghana, 2021). Overall, the Sene East district was ranked the most deprived and least developed district in Ghana.

### Study population and sampling

All teachers in the district constituted the study population, while teachers in government primary schools (primary 1-primary 6) formed the study sample. At the primary school level of children’s development, they form their active stage for lifelong learning and forming attitudes by observing and practising (Peckham, 2016). Hence, the rationale for investigating their school teachers’ attitudes towards HWWS. All 217 primary school teachers in the district were targeted for the study, as there were no time or resource constraints. The essence was also to achieve a larger sample size to make a justifiable generalisation. At the end of the data collection period, 214 teachers participated.

### Data collection methods and procedure

A researcher-administered questionnaire was designed by the research team, taking into consideration the localised nature of the study setting. The questionnaire was subjected to cross-checks by peers, an expert in behavioural research, and subsequent approval by two of our study supervisors. The instrument was then pretested in two primary schools in the Sene West district in order to identify and correct weaknesses and ensure content validity. The questionnaires were administered in English, as all respondents were educated and use English on a daily basis for teaching. The researchers administered the questionnaire so as to ensure that all the respondents adequately understood each question before responding. This helped us obtain reliable and complete data from the respondents. The administration of each questionnaire took an average of 30 min to complete.

The questionnaire contained sub-sections that sought data on the demographic characteristics of the respondents, their reported handwashing practises at critical times – after using the toilet and before eating with the bare hand – their knowledge and attitude toward handwashing with soap, and their perceived susceptibility to and severity of diarrhoea. The respondents’ knowledge of the HWWS was measured using TRUE or FALSE questions, while their attitude, perceived susceptibility to, and severity of diarrhoea were measured on a 5-point Likert scale ranging from strongly disagreeing to strongly agreeing.

### Data analysis

All the investigators screened and cross-examined the collected data for completeness and consistency. The questionnaire was then entered into an Excel file for further cross-checking and subsequently exported to STATA software version 14.0 for analysis. Statistical analysis, including descriptive, Pearson’s correlations, and multiple linear regression, was conducted to determine which variables constituting the various constructs (knowledge, attitude, perceived susceptibility, and perceived severity) predict the teachers’ hand hygiene behaviour. A confidence interval of 95% was used in determining the association between the variables forming each construct and the practise of hand hygiene by the teachers. The raw scores were also estimated using the Praxis test score method. Due to the uniformity of the primary schools in the study area in terms of the availability of WASH infrastructure, the data from the various schools was treated as a homogeneous data. Standard errors were also computed to account for the clustering of the schools.

The dependent variable in this study was the teachers’ reported HWWS practices. The independent variables were knowledge and attitude toward HWWS, perceived susceptibility, and severity of diarrhoea.

### Operational definition of variables

**Reported handwashing practises** represent reported accounts of primary school teachers’ handwashing with soap at critical times – after using the toilet and before eating with the bare hands.

**Knowledge on handwashing with soap** was the level of know-how primary school teachers demonstrated on proper handwashing with soap practices.

**Attitude toward handwashing with soap** represents the level and type of attitude (positive or negative) demonstrated by primary school teachers towards handwashing with soap.

**Perceived Susceptibility to diarrhoea** represents teachers’ perception with regard to their contracting diarrhoea or not.

**The perceived severity of diarrhoea** represents teachers’ perceptions in relation to the seriousness of diarrhoeal diseases.

### Research ethics

Research ethical issues, including informed consent, anonymity, and confidentiality, were adhered to during the study process. Ethical clearance was given by the Committee on Human Research and Publication Ethics (CHRPE) of the Kwame Nkrumah University of Science and Technology (reference number: CHRPE/AP/513/19).

## Results

### Sample characteristics

The respondents’ ages ranged from 20 to 51 years, with an average of 34.7 **± 7.6** years.Those between the ages of 31 and 40 were the majority (40.2%). The majority of the respondents were male (87.9%), and nearly half were single (48.1%), while 45.8% were currently married. The majority of the respondents attained tertiary education (97.6%), while the rest had education up to senior high school level. Most of the respondents were Christians (88.2%) (see Table 1).

**Table 1.** Demographic Characteristics of Respondents.

Characteristics	*n = 214*	%
**Age group**		
20–30years	83	38.8
31–40 years	86	40.2
41–50 years	33	15.4
51+ years	12	5.6
Mean (SD)	34.7 ± 7.6	
**Sex**		
Female	26	12.2
Male	188	87.9
Marital status		
Single	103	48.1
Currently Married	98	45.8
Divorced	13	6.1
**Educational level**		
Senior High School (SHS)	5	2.4
Tertiary	206	97.6
**Religion**		
Christian	187	88.2
Islam	24	11.3
Traditionalist	1	0.5

Source: field data, 2019.

### Bivariate analysis

#### Reported handwashing with soap practices

The respondents’ reported handwashing with soap practices as presented in Table 2 below showed that majority of them (95.8%) affirmed to have visited the toilet within the last 24 h. The standard deviation (SD) for the number of visits was 1. 91 **± **0.7. Three-quarters of those polled had visited the toilet at least once. Most of the respondents reported washing their hands with soap under running water (83.6%; CI 95% 0.56-0.85) after visiting the toilet. The mean ± SD of the handwashing episode was 1.7 ± 0.7. Also, the majority of the respondents reported having eaten with their bare hands within the past 24 h (87.3%), and more than half of them did that between 2–3 times in a day (55.5%). On average, the respondents ate with bare hand 2.9 ± 1.2 times daily. Also, most of the respondents (86.2%; CI 95% 0.2-0.88) reported that they wash their hands when they are about to eat and, on average, they do that by 2.6 ± 1.1 daily as indicated in Table 2 below.

**Table 2.** Reported Handwashing Practices.

Variables	*n = 214*	%
**Visit toilet**		
No	10	4.2
Yes	204	95.8
**Number of visits within the past 24hrs (*n = 204*)**		
1–2 times	158	77.5
3–4 times	46	22.5
**Mean (SD)**	1. 91 **± **0.7	
**Washed hands with soap within the past 24hrs after tiolet use**		
No	34	16.0
Yes	179	84.0
**Handwashing episode within the past 24hrs (*n = 179*)**		
1–2 times	150	84.3
3 times	28	15.7
**Mean (SD)**	1.7 ± 0.7	
**Did you eat with bare hands**		
No	27	12.7
Yes	186	87.3
**Number of times eaten with bare hands for last 24 h (*n = 186*)**		
1 time	27	14.5
2–3 times	104	55.5
4–5 times	55	29.6
**Mean (SD)**	2.9 ± 1.2	
**Do you wash your hands before eating**		
No	42	19.7
Yes	171	80.3
**Episodes of handwashing before eating**		
1 time	37	21.6
2–3 times	96	56.2
4–5 times	38	22.2
**Mean (SD)**	2.6 ± 1.1	

Source: field data, 2019.

#### Relationship between toilet use and reported handwashing

**Figure 1.**
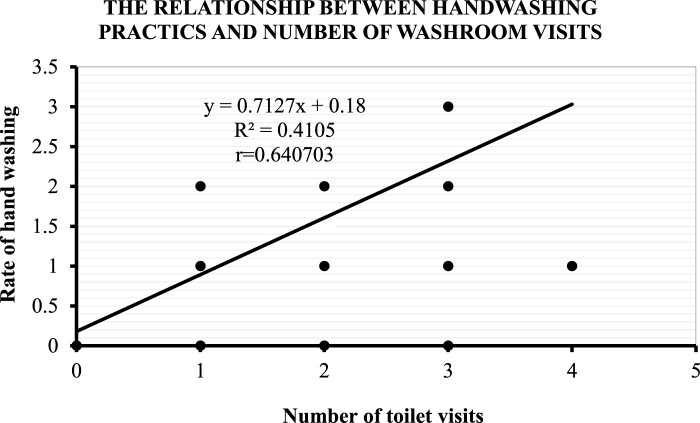
Relationship between Reported Handwashing Behaviour and Toilet Use.

In Figure 1 below, there was a positive correlation between toilet use and reported handwashing among teachers (*r* = 0.64, *p* < 0.001), such that as the number of times a respondent visits the washroom increases, the number of reported handwashing episodes also increases.

#### Relationship between eating with bare hand and reported handwashing

**Figure 2.**
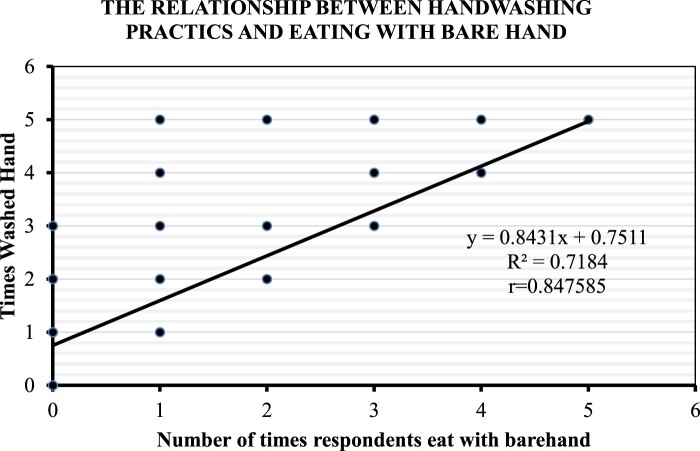
Relationship between Handwashing Practices and Eating with Bare hand.

Similarly in Figure 2 below, there was a positive relationship between eating with the bare hand and reported handwashing among teachers (*r *= 0.84; *p* > 0.001), such that as the number of times a respondent eats with the bare hand increases, the number of reported handwashing episodes also increases.

### Multivariate analysis

#### Respondents’ knowledge on handwashing

Table 3 below presents the respondents’ knowledge on handwashing with soap in basic areas. The majority of them, 193 (90.2%) and 202 (94.4%), knew that hands must be wet before soap application and should be extended to the upper wrist region, respectively. Washing hands in between the fingers was also acknowledged as important by 205 (95.8%) of respondents, while 4.2% thought it was not. Similarly, 196 (91.6%) of the respondents also knew it was not a good practise to wash hands in a basin as shown in Table 3 below. Taken together, 83.9% of respondents had *high knowledge* of handwashing with soap practises for the variables explored in this study.

**Table 3.** Respondents’ Knowledge on Handwashing.

Variable	Frequency (*n* = 214)	Percentage (%)
Soap must be applied before wetting hands		
**True**	21	9.8
**False**	193	90.2
Washing of Upper Wrist Region is Important		
**True**	202	94.4
**False**	12	5.6
Washing in-between the fingers not Important		
**True**	9	4.2
**False**	205	95.8
Hand Washing must atleast 5 min		
**True**	12	5.6
**False**	202	94.4
Washing Hands in a Basin is a Good Practice		
**True**	18	8.4
**False**	196	91.6

Source: field data, 2019.

#### Knowledge of handwashing as a determinant of reported HWWS after using the toilet and before eating with bare hand

Knowledge of HWWS explored in this study statistically predicted reported HWWS behaviours of teachers after using the toilet (*p* < 0.001; *R*^2^*^ ^= *0.111) significantly as shown in Table 4 below. At a significance level of *p* < 0.05, all variables contributed statistically significantly to the prediction. Similarily, knowledge of HWWS explored in the study also statistically significantly predicted reported HWWS behaviours of teachers before eating with bare hands (*p* = 0.002; *R*^2^*^ ^= *0.101). At a significance level of *p* < 0.05, all variables contributed statistically significantly to the prediction as presented in Table 4 below.

**Table 4.** Multiple linear regression analysis of respondents’ knowledge on handwashing and handwashing behaviour after toilet visit and handwashing behaviour before eating with bare hands.

Knowledge on handwashing and handwashing behaviour after toilet visit [Pro > *F* = 0.001]				
Variable	Coeff. (*β*)	Standard error	95% CI	*p*-value
Hand wetting before soap	0.77	0.22	0.33−1.10	0.001
Rubbing in-between fingers	0.76	0.33	0.12–1.41	0.02
Duration of HWWS	0.47	0.28	0.07–1.02	0.03
*R-square*	0.111			
Knowledge on handwashing and handwashing behaviour before eating with bare hands [Pro > *F* = 0.002]				
Variable	Coeff. (*β*)	Standard error	95% CI	*p*-value
Hand wetting before soap	1.10	0.34	0.43 −1.77	0.001
Rubbing in-between fingers	1.11	0.52	0.86–2.14	0.03
Duration of HWWS	1.12	0.43	0.27–1.96	0.01
*R-square*	0.101			

Source: Field data, 2019 Statistic is significant at 0.05 level.

#### Respondents’ attitude to handwashing

Table 5 below presents the attitudes of respondents toward hand washing with soap. Only responses with values are presented for each variable. The majority of respondents 202 (94.8%) strongly disagreed, with only 12 (5.2%) agreeing that hand washing is not important. Similarly, 168 (78.5%) strongly agreed and 43 (20.1%) agreed that hand washing before meals was important. With respect to handwashing being pleasant, 70 (31.7%) of the respondents strongly agreed and 63 (29.5%) agreed with the assertion; while 75 (35.1%) were neutral and 6 (2.8%) of the respondents disagreed. When asked if handwashing wastes time, 51.9% and 35.5% of the respondents, respectively, disagreed and strongly disagreed; 5.1% of them were neutral, 3.7% agreed and another 3.7% strongly agreed with the assertion as presented in Table 5 below.

**Table 5.** Respondents’ Attitude to Handwashing.

Variable	Frequency (*n* = 214)	Percentage (%)
**Hand washing not Important** Strongly Disagree Disagree	202 12	94.8 5.2
**Hand Washing before Meals is Important** Strongly Disagree Disagree Agree Strongly Agree	2 1 43 168	0.9 0.5 20.1 78.5
**Hand Washing is Pleasant** Disagree Neither Agree/Disagree Agree Strongly Agree	6 75 63 70	2.8 35.1 29.4 31.7
**Hand Washing Wastes Time** Strongly Disagree Disagree Neither Agree/Disagree Agree Strongly Agree	76 111 11 8 8	35.5 51.9 5.1 3.7 3.7

Source: field data, 2019.

#### Attitude to handwashing as a determinant of reported HWWS after using the toilet and before eating with bare hand.

Attitude toward HWWS explored in the study statistically significantly predicted reported HWWS behaviours of teachers after using the toilet (*p* < 0.001; *R*^2^*^ ^= *0.077) as indicated in Table 6 below. However, at a significance level of *p* < 0.05, none of the variables added statistically significantly to the prediction, with the exception of teachers’ beliefs that HWWS is time-wasting (*p* = 0.03).

**Table 6.** Multiple linear regression analysis of respondents’ attitude to handwashing and handwashing behaviour after visiting the toilet and before eating with bare hands.

Attitude to handwashing and handwashing behaviour after visiting the toilet [Pro > *F* = 0.0100]				
Variable	Coeff. (*β*)	Standard error	95% CI	*p*-value
Importance of HWWS	−0.56	0.19	−0.42–0.31	0.77
HWWS before meals important	0.22	0.17	−0.12–0.56	0.20
HWWS is pleasant	−0.11	0.08	−0.26–0.04	0.06
HWWS wastes time	0.16	0.07	0.20–0.30	0.03
*R-square*	0.077			
Attitude to handwashing and handwashing behaviour before eating with bare hands [Pro > *F* = 0.002]				
Variable	Coeff. (*β*)	Standard error	95% CI	*p*-Value
Importance of HWWS	0.28	0.28	0.27–0.83	0.32
HWWS before meals important	0.25	0.25	−0.25–0.75	0.33
HWWS is pleasant	−0.03	0.20	−0.42–0.37	0.89
HWWS wastes time	0.32	0.11	0.11–0.53	0.03

Source: Field data, 2019 Statistic is significant at 0.05 level.

Similarly, attitude toward HWWS statistically significantly predicted reported HWWS behaviours of teachers before eating with the bare hand (*p* = 0.002; *R*^2^*^ ^= *0.111). However, all the variables did not add statistically significantly to the prediction at a significance level of *p* < 0.05, except the teachers’ attitude toward “HWWS wastes time,” which added statistically significantly to the prediction (*p* = 0.03) as presented Table 6 below.

#### Teachers’ perceived severity of diarrhoea

Table 7 below presents the distribution of teachers’ perceived severity of diarrhoea. It has it that 164 (76.8%) respondents strongly disagreed that diarrhoea is **not** a serious condition, while 5 (2.4%) strongly agree. Similarly, 119 (55.0%) and 88 (41.7%) of the respondents perceived diarrhoea as a condition that can prevent their school attendance, while a total of 5 (2.5%) of the respondents disagreed. The majority of them also regarded diarrhoea as fatal, except for 7 (3.3%) respondents who believed otherwise as indicated in Table 7 below.

**Table 7.** Teachers’ Perceived Severity of Diarrhoea.

Variable	Frequency (*n* = 214)	Percentage (%)
**Diarrhoea is not a serious condition** Strongly Disagree Disagree Neither Agree/Disagree Agree Strongly Agree	164 42 1 2 5	76.6 19.6 0.5 0.9 2.3
**Diarrhoea could prevent me from going to class** Strongly Disagree Disagree Neither Agree/Disagree Agree Strongly Agree	1 4 2 88 119	0.5 1.9 0.9 41.7 55.0
**Diarrhoea is not fatal** Strongly Disagree Disagree Agree Strongly Agree	147 59 5 2	68.6 28.1 2.4 0.9
**Diarrhoea can’t cause outbreak in school** Strongly Disagree Disagree Neither Agree/Disagree Agree	142 64 4 4	65.9 30.3 1.9 1.9

Source: Author Survey, 2019.

#### Perceived severity of diarrhoea as a determinant of reported HWWS after using the toilet and before eating with bare hand

Teachers’ perceived severity of diarrhoea statistically significantly predicted the reported HWWS behaviours of teachers after using the toilet (*p* = 0.009; *R*^2^*^ ^= *0.086) as presented in Table 8 below. All the individual variables did not add statistical significance to the prediction at a significance level of *p* < 0.05, except one, the perception that “diarrhoea prevents class attendance,” which added statistically significantly to the prediction (*P* = 0.001).

**Table 8.** Multiple linear regression analysis of respondents’ perceived severity of diarrhoea and handwashing behaviour after visiting the toilet and behaviour before eating with bare hands

Perceived severity of diarrhoea and handwashing behaviour after visiting the toilet Pro > *f* = 0.009				
Variable	Coeff. (*β*)	Standard error	95% CI	*p*-value
Diarrhoea not serious	0.16	0.09	−0.27–0.34	0.09
Diarrhoea not fatal	−0.06	0.26	−0.57–0.45	0.81
Diarrhoea can’t cause outbreak	−0.13	0.13	−0.37–0.11	0.37
Diarrhoea prevents class attendance	0.33	0.13	0.59–0.08	0.01
*R-square*	0.086			
Perceived severity of diarrhoea and handwashing behaviour before eating with bare hands Pro > *F* = 0.0016				
Variable	Coeff. (*β*)	Standard error	95% CI	*p*-value
Diarrhoea not serious	0.09	0.16	−0.24–0.41	0.60
Diarrhoea not fatal	−0.26	0.26	−0.78–0.25	0.31
Diarrhoea can’t cause outbreak	−0.25	0.21	−0.67–0.16	0.23
Diarrhoea prevents class attendance	0.38	0.22	−0.85–0.01	0.06

Source: Field data, 2019 Statistic is significant at 0.05 level.

Teachers’ reported HWWS behaviours before eating with bare hands were statistically significantly predicted by their perceived severity of diarrhoea (*p* = 0.002; *R*^2^ = 0.14). However, at a significance level of *p* < 0.05, none of the individual variables added statistical significance to the prediction. (**see** **Table 8** **below**)

#### Teachers’ perceived susceptibility to diarrhoea

The teachers’ perceived susceptibility to diarrhoea, as presented in Table 9 below, showed that, about 96.2% of the teachers believed that failure to wash their hands after visiting the toilet makes them susceptible to diarrhoea diseases, while about 2.8% of them believed otherwise. Similarly, over 98% of the respondents accepted that failure to wash hands before meals could lead to diarrhoea. Concerning the possibility of spreading germs to others due to failure to wash hands at critical times, approximately 92.9% thought it was possible, while 3.8% were undecided.The remaining 3.3% of teachers believed there was no risk of spreading germs to others if they did not wash their hands at critical times (see Table 9 below).

**Table 9.** Teachers’ Perceived Susceptibility to Diarrhoea.

Variable	Frequency (*n* = 214)	Percentage (%)
**Failure to wash hands after toilet could lead to contracting diarrhoea** Strongly Disagree Disagree Neither Agree/Disagree Agree Strongly Agree	1 5 2 30 176	0.5 2.3 0.9 14.0 82.2
**Not washing hands before meals could lead to diarrhoea** Disagree Neither Agree/Disagree Agree Strongly Agree	2 1 47 164	0.9 0.5 22.3 76.3
**I could spread germs if I failed to wash my hands at critical times.** Strongly Disagree Disagree Neither Agree/Disagree Agree Strongly Agree	2 5 8 48 51	0.9 2.4 3.8 22.8 70.1

Source: Field data, 2019.

#### Perceived susceptibility of diarrhoea as a determinant of reported HWWS after visiting the toilet and before eating with bare hand

Based on the teachers’ perceived susceptibility to diarrhoea, a multiple linear regression analysis was used to predict their handwashing behaviours before eating with bare hands.The variable failed to statistically predict HWWS behaviours of teachers before eating with bare hands (*P* < 0.055; *R*^2^*^ ^= 0.064)*. And none of the variables add statistically significance to the prediction (*P* < 0.05), as indicated in Table 10 below.

**Table 10.** Multiple linear regression analysis of respondents’ perceived susceptibility to diarrhoea and handwashing behaviour after visiting the toilet and before eating with bare hands.

Perceived susceptibility to diarrhoea and handwashing behaviour after visiting the toilet [Pro > *F* = 0.0323]				
Variable	Coeff. (*β*)	Standard error	95% CI	*p*-value
Not washing hands after toilet	0.37	0.24	−0.85–0.10	0.12
Not washing hands before meals	0.06	0.15	−0.23–0.35	0.68
Spreading germs after toilet	0.01	0.12	−0.23–0.25	0.94
*R-square*	0.064			
Perceived susceptibility to diarrhoea and handwashing behaviour before eating with bare hands [Pro > *F* = 0.0690]				
Variable	Coeff. (*β*)	Standard error	95% CI	*p*-value
Not washing hands after toilet	0.44	0.36	−1.15–0.28	0.23
Not washing hands before meals	0.03	0.22	−0.41–0.46	0.91
Spreading germs after toilet	0.25	0.19	−0.12–0.61	0.91
*R-square*	0.055			

Source: *Field data, 2019* Statistic is significant at 0.05 level.

Also, Table 10 below shows a multiple linear regression analysis to predict handwashing behaviours of teachers after visiting the toilet based on the teachers’ perceived severity of diarrhoea. The variable statistically significantly predicted HWWS behaviours of teachers after using the toilet (*P* < 0.009; *R*^2^*^ ^= *0.086). All the individual variables did not add statistical significance to the prediction (*P* < 0.05), except one, as indicated in Table 10.

## Discussion

### Reported handwashing with soap

Measuring social behaviours such as hygiene can be challenging due to people’s inclination to change their behaviour when observed or overreport when asked (Biran et al., 2008). Even though the self-reported nature of this study’s data posed issues of social desirability bias in the participants’ responses (UNICEF, 2013), extending Subar et al.'s (2015) response to criticisms against self-reported dietary data suggests that a precise measurement is not required for self-reported data to be useful for informing policy and designing interventions for a desired outcome. Therefore, to guide people toward positive hand hygiene practices, asking them about their current practices or behaviours is important and should not be underestimated (ibid). Previous self-reported studies on handwashing behaviour or practises conducted by Cui et al. (2021) among chefs in China's Jiangsu Province, Elkhawaga and El-Masry (2017) among Egyptian medical students, and Cruz and Bashtawi (2015) among Saudi nursing students found their findings relevant for initiating interventions aimed at improving handwashing knowledge and practises among their study participants.This gave an indication that self-reported data can be much more credible when the consent process appropriately builds trust among the participants compared to data obtained through observation, as participants have the tendency to change their typical behaviour, especially when they notice that they are being observed (Biran et al., 2008). The results of this study can therefore be used as the basis for instituting hand hygiene intervention programmes in the Sene East and other rural districts of Ghana.

The reported practise of hand washing with soap (HWWS) in this study was found to be encouraging among primary school teachers at critical times – after toilet use (84.0%) and before eating with the bare hands (80.3%). This is supported by Shmidt et al.'s (2009) study where there was more compliance with hand hygiene practices after feacal contact than before eating with bare hand. Although this study did not investigate participants’ motivations for practising HWWS, the perceived risk of contaminating hands with faecal bacteria after toilet use may have accounted for the relatively higher proportion of participants washing their hands after toilet use compared to handwashing before eating with bare hands, partly because, public toilets are generally open and filthy in rural communities, including school settings in Ghana (Peprah et al., 2015). The study by Burton et al. (2011) confirmed that faecal bacteria are the most common bacteria on contaminated hands.In general, the level of handwashing practises reported in this study was higher than that reported by Hirai et al. (2016) among Indonesian households (approximately 70%) and De-Alwis et al. (2012) among medical students after toilet use.

Increased activities such as using the toilet and eating with bare hands correlated positively with reported handwashing with soap among primary school teachers in the Sene East district. This resonates with Vivas *et al.'s* (2011) report of a positive association between reported handwashing with soap and eating with the bare hand among pupils in Ethiopia who also showed a high rate of handwashing. Appiah-Brempog et al.'s (2017) study showed that the most common approach to handwashing in the school environment is handwashing with soap. Despite the high rate of handwashing at critical times among teachers, it was surprising that approximately 20% of them reported eating with their bare hands without handwashing and that approximately 16% also use the toilet without handwashing, despite their high educational level. Hirai et al. (2016) studies showed that adequate water availability is a vital precursor to performing handwashing with soap, but no significant association was established through their multivariate analysis. Although this study did not specifically investigate water availability, the behaviour of some teachers has serious consequences for the type and nature of handwashing attitude that students will imitate from them.

#### Determinants of handwashing behaviour among teachers

The teachers’ knowledge of HWWS parameters and their reported handwashing with soap behaviours after toilet use and before eating with bare hands were found to be statistically significant (*p* < 0.001 and *p* = 0.002 respectively). These findings could be interpreted to suggest that knowledge of HWWS is an important determining factor for primary school teachers to observe handwashing with soap at critical times – before eating with the bare hands and after visiting the toilet, and supported by White et al. (2020), Chittleborough et al. (2013) and the Global Handwashing Partnership (2018). Knowledge of hand hygiene and its practise significantly reduces the chances of illness and absenteeism in schools (Sohn et al., 2012).

Similarly, the teachers’ attitude towards HWWS practises also predicted their hand washing behaviours at critical times. The present study found statistical significance between teachers’ attitudes towards HWWS and HHWS behaviour at critical times: after using the toilet (*p* < 0.001; *R*^2^*^ ^= *0.077) and before eating with the bare hand (*p* = 0.002; *R*^2^*^ ^= *0.111). At both critical periods, only the participants who believed that HWWS is time-wasting showed statistical significance (*p* = 0.03). Aledeilah et al. (2018) found attitudes of this nature toward handwashing, which subsequently translated into actual handwashing practices. Perhaps citing HWWS as a waste of time limited teachers’ use of HWWS at critical times.

This study’s results also tend to support Park et al.'s (2010) findings that where people perceive the severity of illness to be greater, compliance with preventive measures is improved. Handwashing behaviours after toilet use (*p* = 0.009) and before eating with bare hands (*p* = 0.0016) were statistically significant with the teachers’ perceived severity of diarrhoea. The teachers’ fear of missing classes due to diarrhoea (*P* = 0.01) appears to be a predicting factor for their handwashing behaviour after toilet use.

In summary, knowledge, attitude toward handwashing with soap, and perceived susceptibility and severity of diarrhoea were factors that appeared to influence the teachers’ reported handwashing with soap behaviours at critical times. The findings of this study have implications for hand hygiene interventions and campaigns. First, health-promoting messages about handwashing and hygiene should be widely disseminated to improve the knowledge of the target population. Second, hand hygiene campaigns should emphasise the benefits of washing hands at critical times and the potential dangers associated with neglecting this simple yet effective intervention to engender positive attitudes and risk perceptions.

#### Study limitations

As earlier noted by Elkhawaga and El-Masry (2017), this study collected self-reported data which made the results not only somewhat subjective, but could also be overreported. However, this was managed by appropriately seeking the respondents’ consent for trust and objectivity in their responses as much as possible. Also, data were not collected on the availability of WASH facilities, even though their presence or absence in the schools could influence the teachers’ practises and attitudes toward hand washing.

Another limitation of the study relates to the possible effect of clustering. Our study sought to recruit all teachers in the selected schools within the study area which decision could have led to the reduction of variability in the responses from participants in the same cluster (school). Since the study did not account for clustering, the interpretation of the findings should be approached in light of the clustering effect phenomenon. Finally, data were collected from one district that was specifically targeted as a resource-deprived district and therefore, the results may not represent all districts in Ghana. However, this study provided useful data that could inform the design of WASH intervention programmes by targeting specific variables constituting the constructs used in this study.

## Data Availability

The data associated with this manuscript is available with the corresponding author on a reasonable request.
